# Serum Fatty Acid-Binding Protein 4 Is a Predictor of Cardiovascular Events in End-Stage Renal Disease

**DOI:** 10.1371/journal.pone.0027356

**Published:** 2011-11-10

**Authors:** Masato Furuhashi, Shutaro Ishimura, Hideki Ota, Manabu Hayashi, Takahiro Nishitani, Marenao Tanaka, Hideaki Yoshida, Kazuaki Shimamoto, Gökhan S. Hotamisligil, Tetsuji Miura

**Affiliations:** 1 Second Department of Internal Medicine, Sapporo Medical University School of Medicine, Sapporo, Japan; 2 Second Department of Internal Medicine, Obihiro Kosei Hospital, Obihiro, Japan; 3 Obihiro East Medical and Cardiovascular Clinic, Obihiro, Japan; 4 Sapporo Medical University, Sapporo, Japan; 5 Department of Genetics and Complex Diseases, Harvard School of Public Health, Boston, Massachusetts, United States of America; Maastricht University, The Netherlands

## Abstract

**Background:**

Fatty acid-binding protein 4 (FABP4/A-FABP/aP2), a lipid chaperone, is expressed in both adipocytes and macrophages. Recent studies have shown that FABP4 is secreted from adipocytes and that FABP4 level is associated with obesity, insulin resistance, and atherosclerosis. However, little is known about the impact of FABP4 concentrations on prognosis. We tested the hypothesis that FABP4 level predicts prognosis of patients with end-stage renal disease (ESRD), a group at high risk for atherosclerosis-associated morbidity and mortality.

**Methods and Results:**

Biochemical markers including FABP4 were determined in 61 ESRD patients on chronic hemodialysis (HD). Serum FABP4 level in females (404.2±30.5 ng/ml) was significantly higher than that in males (315.8±30.0 ng/ml), and the levels in ESRD patients were about 20-times higher than those in age-, gender- and body mass index (BMI)-matched control subjects with normal renal function. FABP4 level was decreased by 57.2% after HD and was positively correlated with blood pressure, BMI, and levels of lipids and insulin. Multiple regression analysis indicated that HD duration, BMI, and triglycerides level were independent determinants for FABP4 level. ESRD patients with high FABP4 levels had higher cardiovascular mortality during the 7-year follow-up period. Cox proportional hazard regression analysis showed that logarithmically transformed FABP4 level was an independent predictor of cardiovascular death adjusted for age, gender, HD duration, BMI, and triglycerides level (hazard ratio, 7.75; 95% CI, 1.05–25.31).

**Conclusion:**

These findings suggest that FABP4 level, being related to adiposity and metabolic disorders, is a novel predictor of cardiovascular mortality in ESRD.

## Introduction

Intracellular lipid chaperones known as fatty acid-binding proteins (FABPs) are a group of molecules that coordinate lipid responses in cells [1]. FABPs are abundantly expressed 14–15 kDa proteins that can reversibly bind to hydrophobic ligands, such as saturated and unsaturated long chain fatty acids, eicosanoids, and other lipids, with high affinity[1]. FABPs have been proposed to facilitate the transport of lipids to specific compartments in the cell, such as to the lipid droplet for storage, to the endoplasmic reticulum for signaling, trafficking, and membrane synthesis, to the mitochondria or peroxisome for oxidation, to cytosolic or other enzymes to regulate their activity, and to the nucleus for lipid-mediated transcriptional regulation. One of the FABPs, fatty acid-binding protein 4 (FABP4), known as adipocyte FABP (A-FABP) or aP2, is expressed in both adipocytes and macrophages and plays important roles in the regulation of insulin sensitivity and the development of atherosclerosis [2]–[8]. Therefore, it is expected that modification of FABP4 function will provide a new class of therapeutic agents. In fact, we recently demonstrated that chemical inhibition of FABP4 could be a therapeutic strategy against insulin resistance, diabetes mellitus (DM), fatty liver disease, and atherosclerosis in experimental models[9].

In the present study, we hypothesized that serum FABP4 is a novel marker for risk stratification of end-stage renal disease (ESRD) patients on hemodialysis (HD). The rationale for this hypothesis is four-fold. First, it has been shown that FABP4 is secreted from adipocytes [10], although there is no typical sequence of secretory signal peptides. At least, we previously confirmed that FABP4 release from adipocytes was not an escape due to apoptosis or necrosis of adipocytes [7]. Second, recent studies have shown that elevation of serum FABP4 is associated with obesity and insulin resistance, risk factors of atherosclerosis, and carotid atherosclerosis [10]–[13]. Third, chronic kidney disease has been shown to be a risk factor of atherosclerosis [14]. Fourth, atherosclerotic vascular diseases are a major cause of death in ESRD patients [14].

## Methods

### Ethics statement

This study was performed with the approval of the institutional ethical committee of Sapporo Medical University, and written informed consent was received from all of the subjects.

### Participants

Sixty-one HD patients (31 males and 30 females) aged 39–78 years (mean age: 61.6±1.8 years, mean±SEM) were recruited. All of the patients had anuria or oliguria (urine volume <200 ml/day). None of the patients had a history of acute myocardial infarction within 6 months prior to the start of this study, chest pain at rest or in the peridialysis period, unstable hemodynamics during dialysis, critical illness, or a history of recent major vascular surgery. The mean duration of HD was 63.4±7.1 months. The underlying renal diseases of the HD patients were diabetes mellitus (n = 21), glomerulonephritis (n = 22), nephrosclerosis (n = 6) and other diseases (n = 12). Diabetic patients treated with thiazolidinediones, PPARγ agonists, were excluded because the FABP4 gene is known as a target of PPARγ. All HD patients were dialyzed 2 or 3 times per week on Monday-Wednesday–Friday, Tuesday–Thursday–Saturday, or Tuesday–Satur day using high-flux membranes (dialysis filter surface area, 1.1– 2.1 m^2^). Sixty-one age-, gender- and body mass index-matched control subjects (31 men and 30 women; mean age, 61.2±2.0 years) who had been taking no medication were also enrolled to compare FABP4 levels between subjects with and without renal dysfunction. None of the control subjects had any evidence of complications such as endocrine or metabolic disturbances, cerebrovascular or cardiovascular disease, and renal disease.

### Measurement of biochemical markers

Blood samples in HD patients were drawn before the start and at the end of a routine HD treatment on Monday or Tuesday. Assays of FABP4 (ng/ml) were per­formed using blood samples taken before and after HD. Other biochemical assays, such as assays of total protein, creatinine, blood urea nitrogen, lipid variables, glucose, insulin, and adiponectin, were performed using blood samples taken before hemodi­alysis. Serum FABP4 level was measured using a commercially available enzyme-linked immunosorbent assay kit (Biovendor R&D, Mordrice, Czech Republic). The accu­racy, precision and reproducibility of this kit have been described previously [10]. Plasma glucose was determined by the glucose oxidase method. Plasma insulin was measured by a radioimmunoassay method (Insulin RIA bead, Dianabot, Tokyo, Japan). Serum lipid profiles, including total cholesterol, high-density lipoprotein (HDL)-cholesterol and triglycerides, were estimated by enzymatic methods. Serum adiponectin level was measured using a commercially available sandwich enzyme-linked immunosorbent assay kit (Otsuka Pharmaceuticals Co., Ltd., Tokushima, Japan).

### Statistical analysis

Numeric variables are expressed as means±SEM. The Mann-Whitney U test was used for comparisons between two unpaired variables. The difference between two paired variables was analyzed by the Wilcoxon signed rank test. Group statistical comparisons were assessed by the chi-square test. Before performing regression analyses, the distribution of each parameter was tested for normality using the Shapiro-Wilk W test, and non-normally distributed parameters were logarithmically transformed. Simple linear regression analysis was used to determine the correlation between two variables. Multiple linear regression analysis was used to identify independent determinants of the FABP4 concentrations and the percentage of variance in the FABP4 concentrations that they explained (R^2^). Receiver operating characteristic (ROC) analysis was performed to determine the inflection point at which the FABP4 level provided the most sensitive prediction of cardiovascular death as a result of sudden cardiac death/arrhythmia, heart failure, atherothrombotic events (myocardial infarction, stroke, mesenteric ischemia). For a given FABP4 level, the ordinate value shows the percentage of patients with that FABP4 level who died (true-positive rate or sensitivity), and the abscissa value shows the percentage of patients with that FABP4 level who did not die (false-positive rate or 1-specificty). Using the ROC-derived optimal cutoff value, patients were divided into two groups. Survival rate was analyzed by log-rank tests of Kaplan-Meier curves in the two groups. Cox regression analyses were performed for cardiovascular death as an end point. A p value of <0.05 was considered statistically significant. All data were analyzed by using JMP 8 for Macintosh (SAS Institute, Cary, NC).

## Results

### FABP4 levels in hemodialysis patients

**Figure 1:**
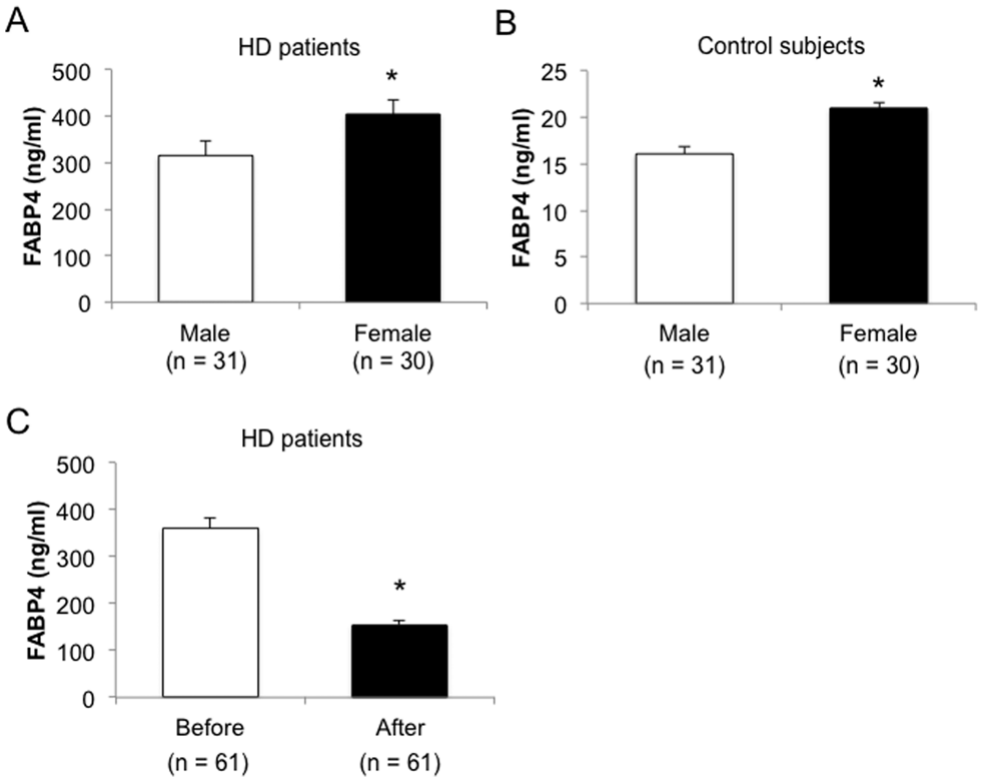
Concentrations of FABP4. FABP4 levels were investigated in the males and females of hemodialysis patients (A) and control subjects with normal renal function (B). FABP4 levels were determined before and after hemodialysis (C). Values are presented as means±SEM. *P <0.05.

The FABP4 level in HD males were significantly lower than that in HD females (Figure 1A; males vs. females: 315.8±30.0 vs. 404.2±30.5 ng/ml), and these levels were about 20-times higher than those in both male and female controls with normal renal function (Figure 1B; males vs. females: 16.0±1.7 vs. 20.5±1.6 ng/ml). After HD, the FABP4 level was significantly decreased by 57.2% (before vs. after: 359.3±22.0 vs. 153.9±10.5 ng/ml) but was still higher than that in control subjects (Figure 1C).

### Simple and multiple regression analyses for FABP4

In all of the HD patients, FABP4 levels were positively correlated with HD duration, body mass index, waist circumference, blood pressures, and levels of total cholesterol, LDL-cholesterol, triglycerides, and insulin and were negatively correlated with levels of HDL-cholesterol and adiponectin (Table 1). Multiple regression analysis for predicting FABP4 level was performed using the correlated and nonconfounding parameters, such as age, gender, HD duration, body mass index as an index of adiposity, systolic blood pressure, and levels of triglycerides as a lipid profile, insulin, and adiponectin. The result revealed that HD duration, body mass index, and level of triglycerides were independent predictors for FABP4 level, explaining a total of 55% of the variance in this measure (R^2^ = 0.55) (Table 2).

**Table 1:** Simple regression analysis for log FABP4.

	r	p
Age	−0.175	0.178
HD duration	0.253	0.049
Body mass index	0.315	0.013
Waist circumference	0.345	0.012
Systolic blood pressure	0.259	0.043
Diastolic blood pressure	0.325	0.011
Total cholesterol	0.378	0.004
LDL cholesterol	0.291	0.024
HDL cholesterol	−0.256	0.046
Triglycerides	0.506	<0.001
Insulin	0.430	0.002
Adiponectin	−0.366	0.004

**Table 2:** Multiple regression analysis for log FABP4.

	t	p
Age	0.118	0.907
Gender (Male)	−1.280	0.207
HD duration	4.215	<0.001
Body mass index	2.320	0.025
Systolic blood pressure	0.192	0.849
Triglycerides	3.277	0.002
Insulin	0.563	0.576
Adiponectin	−1.651	0.106
R^2^ = 0.55		

### Predictor of cardiovascular mortality in ESRD

**Figure 2:**
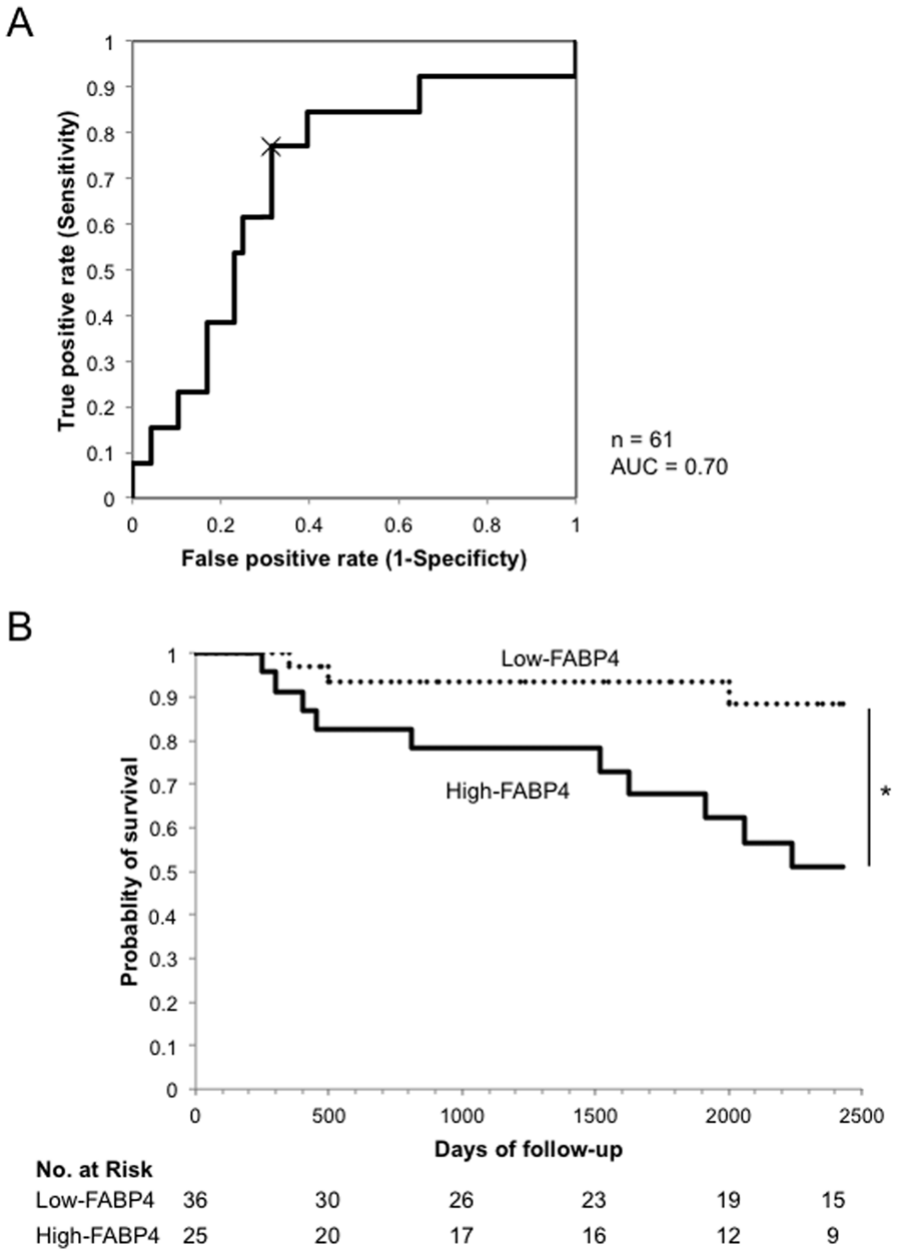
Receiver operating characteristic (ROC) analysis and Kaplan-Meier survival curve. (A) ROC analysis of the correlation between FABP4 levels in HD patients and the occurrence of cardiovascular death is shown. The ordinate values show the corresponding true-positive rate (fraction of patients with that FABP4 level who died) and the abscissa values show the corresponding false-positive rate (fraction of patients with that FABP4 level who did not die). The inflection point (indicated by X) was chosen as the optimal cutoff value, 364.1 ng/ml (sensitivity: 76.9%, specificity: 68.8%, area under the curve [AUC]: 0.70). (B) According to the cutoff point of FABP4 level, HD patients were divided into two groups: Low-FABP4 and High-FABP4 groups, and Kaplan-Meier curves for cardiovascular death are analyzed in the two groups. Broken lines (Low-FABP4 group), patients with FABP4 levels of <364.1 ng/ml (n = 36, 22 males and 14 females); Solid lines (High-FABP4 group), patients with FABP4 levels of ≥364.1 ng/ml (n = 25, 9 males and 16 females). Survival rates were compared by log-rank test. *P <0.05.

During the 7-year follow-up period, we confirmed 13 out of 61 patients who died of cardiovascular events. ROC analysis of the correlation between FABP4 levels in HD patients and the occurrence of cardiovascular death was performed. We chose the inflection point as the optimal cutoff value, 364.1 ng/ml (Figure 2A; sensitivity: 76.9%, specificity: 68.8%, area under the curve: 0.70). Using the cutoff point, HD patients were divided into two groups: Low-FABP4 (HD patients with FABP4 levels below the point) and High-FABP4 (HD patients with FABP4 levels above the cutoff) groups. During the 7-year follow-up period, 3 out of 36 Low-FABP4 patients and 10 out of 25 High-FABP4 patients died of cardiovascular events. Kaplan-Meier survival curves showed that the High-FABP4 patients had a significantly higher mortality than did the Low-FABP4 patients (Figure 2B).

Clinical characteristics of the HD patients split by the two groups are shown in Table 3. There were no significant inter-group differences in age, gender, presence of DM, blood pressure, and Kt/V as an index of dialysis adequacy. Drug therapies including administration of antihypertensive drugs and statins were comparable in the Low-FABP4 and High-FABP4 groups. Body mass index and waist circumference were significantly higher in the High-FABP4 patients than in the Low-FABP4 patients. The High-FABP4 patients had significantly higher levels of total cholesterol, triglycerides, and insulin and lower levels of adiponectin than did the Low-FABP4 patients. No significant differences were found in other parameters, including serum total protein, blood urea nitrogen, creatinine, potassium, and C-reactive protein, between the Low-FABP4 and High-FABP4 groups.

**Table 3:** Basal and biochemical characteristics of 61 patients.

	Whole	Low-FABP4	High-FABP4
n	61	36	25
Male/Female	31/30	22/14	9/16
Age (years)	61.6±1.8	62.6±2.3	60.2±2.8
Diagnosis			
Diabetes mellitus	21	12	9
Glomerulonephritis	22	10	12
Nephrosclerosis	6	6	0
Others	12	8	4
HD duration (months)	63.4±7.1	51.4±9.0	80.6±10.8*
Body mass index (kg/m^2^)	21.4±0.5	20.7±0.6	22.4±0.4*
Waistcircumference (cm)	78.0±1.5	75.3±2.0	81.4±2.3*
Systolic blood pressure (mmHg)	140.0±2.9	137.4±3.8	143.7±4.5
Diastolic blood pressure (mmHg)	77.0±1.8	74.0±2.3	80.4±2.7
Kt/V	1.44±0.05	1.42±0.06	1.45±0.08
Drug therapy			
ACE inhibitors	5 (8.2)	2 (5.6)	3 (12.0)
Angiotensin II receptor blockers	16 (26.2)	11 (30.5)	5 (20.0)
Calcium channel blockers	26 (42.6)	18 (50.0)	8 (32.0)
α blockers	4 (6.6)	4 (11.1)	0 (0)
β blockers	7 (11.5)	4 (11.1)	3 (12.0)
Diuretics	13 (21.3)	8 (22.2)	5 (20.0)
Statins	8 (13.1)	4 (11.1)	4 (16.0)
Total protein (g/l)	6.4±0.1	6.4±0.1	6.4±0.1
BUN (mg/dl)	63.4±2.4	62.4±3.1	64.9±3.8
Cr (mg/dl)	10.1±0.3	9.7±0.4	10.6±0.5
K (mEq/l)	5.3±0.1	5.2±0.1	5.5±0.2
Total cholesterol (mg/dl)	168.6±4.7	159.4±5.9	181.7±7.0*
HDL cholesterol (mg/dl)	40.9±1.7	43.5±2.2	37.2±2.6
LDL cholesterol (mg/dl)	101.5±3.6	96.2±4.6	109.4±5.6
Triglycerides (mg/dl)	112.5±7.2	90.2±8.2	142.7±9.7**
Blood glucose (mg/dl)	124.7±6.4	115.4±8.2	137.5±9.7
Insulin (µU/ml)	20.4±2.4	15.7±3.1	27.0±3.6*
HbA1c (%)	5.4±0.2	5.2±0.3	5.6±0.3
C-reactive protein (mg/dl)	0.45±0.08	0.44±0.10	0.47±0.12
Adiponectin (µg/ml)	17.7±1.0	19.5±1.3	15.0±1.6*

Variables are expressed as means±SEM or number (%).

*P <0.05 vs. Low-FABP4.

**P <0.01 vs. Low-FABP4.

Cox proportional hazard regression analysis for predicting cardiovascular death was performed including FABP4 level, age, gender, presence of DM, and the independent determinants of FABP4 level (HD duration, body mass index, and level of triglycerides) in the multiple regression analysis (Table 2) which also have a significant difference between the Low-FABP4 and High FABP4 groups (Table 3). The result demonstrated that FABP4 concentration was a significant explanatory variable after adjustment of the other parameters, suggesting that FABP4 level was an independent predictor of long-term cardiovascular mortality (Table 4).

**Table 4:** Cox proportional hazard model for prognosis at 7 year follow-up.

	HR	95% CI	p
Age	1.054	1.005 – 1.117	0.029
Gender (Male)	2.447	0.715 – 9.177	0.153
Diabetes mellitus	1.198	0.317 – 4.390	0.783
HD duration	0.993	0.978 – 1.006	0.293
Body mass index	0.866	0.678 – 1.079	0.209
log Triglycerides	0.881	0.135 – 6.511	0.898
log FABP4	7.751	1.052 – 25.316	0.044

## Discussion

To the best of our knowledge, this is the first report on the impact of elevation of circulating FABP4 concentrations on long-term prognosis of ESRD. Previous studies using animal models indicate that FABP4 plays a significant role in several aspects of metabolic syndrome, including insulin resistance, type 2 DM and atherosclerosis, through its action at the interface of metabolic and inflammatory pathways in adipocytes and macrophages [1]–[8]. In a human study, reduced expression of FABP4 in adipose tissue was suggested to have beneficial effects on cardiovascular and metabolic health [15]. Subjects with a genetic variation of the FABP4 locus (T-87C) leading to decreased FABP4 expression in adipose tissue showed lower levels of triglycerides as well as a significantly reduced risk for cardiovascular disease. Taken together, these observations support the notion that measurement of FABP4 concentration is useful for risk stratification and for guiding treatment in observational or interventional studies. However, it is clearly necessary to prospectively evaluate whether a change in the FABP4 value indeed reflects conditions of metabolic syndrome and atherosclerosis and predicts long-term cardiovascular outcomes in patients regardless of ESRD.

In the present study, FABP4 concentrations were sex-related, being higher in females than in males as previously reported [10]–[13], [16]. However, multiple regression analysis showed that gender was not a significant determinant of FABP4 concentration in HD patients. FABP4 level was significantly higher in HD patients than in the controls, which is consistent with previous findings in patients with renal dysfunction [16]–[18]. In the control subjects, FABP4 levels were negatively correlated with estimated glomerular filtration rate (data not shown), which is an index of renal function, as previously reported in DM patients with nephropathy [16]. We previously measured concentrations of FABP3, which is another member of the FABP family known as heart-type FABP (H-FABP), in HD patients [19]. We found the concentration of FABP3 was increased 13.8-fold in HD patients compared to that in control subjects with normal renal function and was reduced by 40% after HD but was still higher than that in control subjects. Those results are similar to the results of the present study. The molecular weights of FABP3 and FABP4 are almost the same, about 15 kD. It is expected that the sieving effects of HD dialyzers on these two proteins are similar. These findings suggest that renal elimination is a major route by which physiological levels of FABPs are maintained. Interestingly, similar mechanisms of elimination have been proposed for other adipocyte-derived factors, such as leptin, adiponectin, and retinol-binding protein 4 [20]–[22]. Taking these into consideration, FABP4 appears to be accumulated in circulation due to diminished renal excretion in chronic kidney disease.

Recent studies have demonstrated an association between increased FABP4 levels and metabolic parameters even in HD patients [17], [18]. In the present study, we confirmed that FABP4 levels were significantly correlated with adiposity, blood pressure, insulin resistance, and dyslipidemia in HD patients. Furthermore, body mass index and triglycerides were independent predictors for FABP4 concentration, and this relationship was independent of HD duration, suggesting that a high level of FABP4 is attributable to metabolic syndrome even in patients with ESRD. Strikingly, FABP4 level was an independent predictor of cardiovascular death after adjustment of metabolic parameters.

One limitation in this study is the small number of patients enrolled. As another limitation, we did not directly assess the extent of atherosclerosis in each patient. Thus, the relationship between FABP4 level and progression of atherosclerosis remains unclear. These issues warrant further investigation in a prospective study recruiting a larger number of patients.

In conclusion, concentration of serum FABP4 may be not only a marker of metabolic syndrome that can be used even for ESRD patients but also a novel predictor of cardiovascular mortality in patients at high risk of atherosclerotic cardiovascular events.
